# Factors influencing implementation of a self-measured blood pressure program in community health centers: an implementation mapping approach

**DOI:** 10.3389/fpubh.2025.1485343

**Published:** 2025-07-17

**Authors:** Serena A. Rodriguez, Fernanda Velasco-Huerta, Mahalia Sampson-Ansah, Ella R. Garza, William B. Perkison, Patenne D. Mathews, Catherine Pulicken, Maria E. Fernandez

**Affiliations:** ^1^Department of Health Promotion and Behavioral Sciences, The University of Texas Health Science Center (UTHealth) at Houston School of Public Health, Dallas, TX, United States; ^2^Center for Health Promotion and Prevention Research, UTHealth Houston, Houston, TX, United States; ^3^Institute for Implementation Science, UTHealth Houston, Dallas, TX, United States; ^4^Department of Health Promotion and Behavioral Sciences, UTHealth Houston School of Public Health, Houston, TX, United States; ^5^Department of Epidemiology, Human Genetics, and Environmental Sciences, UTHealth Houston School of Public Health, Houston, TX, United States

**Keywords:** implementation mapping, health promotion, hypertension, self-measured blood pressure, remote patient monitoring, program planning

## Abstract

**Objectives:**

Uncontrolled hypertension is a leading cause of cardiovascular disease, particularly among adults aged 45 years and older. Self-measured blood pressure (SMBP) is an evidence-based intervention that can help patients manage hypertension outside of the clinical setting. We conducted a needs and assets assessment to identify (1) health center adopters and implementers and (2) barriers and facilitators to SMBP adoption and implementation in six community health centers in Texas.

**Methods:**

Data sources included: (1) needs and assets assessment surveys and semi-structured interviews; (2) site visits with participating health centers; and (3) detailed meeting notes and logs. Leaders and administrators from the participating health centers completed a self-administered 56-item survey. We computed descriptive statistics for survey data. For open-ended survey responses, interview data, and meeting notes, team members labeled the reported and observed barriers and facilitators to program implementation.

**Results:**

Barriers to SMBP adoption and implementation included staffing shortages, limited funding to procure blood pressure devices, and perceived challenges reaching patients and maintaining engagement in an SMBP program. Facilitators included existing hypertension management guidelines, health center familiarity with SMBP programs, and the use of non-physician team members in hypertension management programs. Adopters included leadership professionals and administrators, and implementers included healthcare providers, and non-physician team members.

**Conclusion:**

Findings inform our understanding of SMBP program adoption, implementation, and importantly, how to best allocate resources to incorporate SMBP programs into clinical workflows.

## Introduction

Approximately 115 million individuals have hypertension in the United States (1, 2). Hypertension is associated with an increased risk of adverse cardiovascular outcomes, such as coronary heart disease, heart failure, stroke, and myocardial infarction, and an increased risk of mortality (3). Self-measured blood pressure (SMBP) is an evidence-based intervention that can help patients manage hypertension outside of the clinical setting (4–8). SMBP includes patients self-measuring their blood pressure at home with clinical support tools that provide clinicians with information about patients’ daily blood pressure (7). SMBP has been shown to improve hypertension treatment adherence and blood pressure outcomes compared to standard care by encouraging patients to take an active role in their healthcare (6, 9–11). However, adoption and implementation of SMBP in community health centers is limited (12).

This study aimed to (1) identify barriers and facilitators to SMBP adoption and implementation and (2) identify program adopters and implementers (i.e., individuals responsible for adopting and implementing SMBP policies and practices) in community health centers serving patient populations that are predominantly under- or uninsured racial and ethnic minorities. Findings will inform the development, implementation, and testing of strategies to increase the adoption and implementation of SMBP in health centers.

## Methods

This study is part of a larger multi-site implementation study promoting the adoption and implementation of evidence-based interventions for hypertension prevention, treatment, and management in community health centers. We used Implementation Mapping, a systematic five-task process for developing, implementing, and evaluating implementation strategies, to increase uptake of SMBP (13). This study reports findings from Implementation Mapping task one, conducting a needs and assets assessment. The University of Texas Health Sciences Center Houston Committee for the Protection of Human Subjects approved all study procedures.

### Sample

This study was conducted in six federally qualified health centers (FQHC) in the Texas Department of State Health Services Public Health Region 6/5 South. This Public Health Region includes 16 counties surrounding the Greater Houston area: Austin, Brazoria, Chambers, Colorado, Fort Bend, Galveston, Harris, Hardin, Jefferson, Liberty, Matagorda, Montgomery, Orange, Walker, Waller, and Wharton counties. We identified health centers serving uninsured or underinsured populations using the HRSA.gov database and contacted the centers’ leadership to introduce the study, discuss the health center’s patient population, and discuss hypertension management needs. Health centers were recruited as part of a larger diabetes and heart disease prevention grant, and health centers indicated during the recruitment process whether or not they would like to participate in SMBP-related activities. Leaders interested in addressing their patients’ needs through SMBP were invited to participate. Health centers were recruited on a rolling basis from 2020 to 2021. Throughout the course of the COVID-19 pandemic, health centers were invited to participate virtually through e-mail and phone communications. Semi-structured interviews and most site visits were conducted via telecommunications platforms throughout most of the COVID-19 pandemic to meet social distancing guidelines. A select few in-person site visits were conducted when deemed safe to do so and with appropriate masking and social distancing.

### Data collection

Data sources included: (1) surveys and follow-up interviews; (2) site visits; and (3) detailed meeting notes and logs. Leaders and administrators from each community health center selected one individual to complete a self-administered 56-item survey asking about patient demographics, electronic health records (EHR) system specifications, level of patient engagement with the EHR, hypertension screening policies and practices, and referral processes to lifestyle change programs targeting hypertension. One survey was completed per community health center. Surveys were conducted using Qualtrics, a HIPAA-compliant web-based survey platform. We conducted 45-60-min semi-structured interviews after survey completion to gain further insight about leaders’ answers. Interviewers captured detailed notes during the interviews; they were not recorded.

Finally, we conducted site visits at the main site of each health center to understand staff roles and responsibilities, identify workflows and processes, and interface with EHR systems. We maintained detailed field notes during and after each visit capturing potential barriers and facilitators to SMBP program adoption and implementation. We sustained a monthly meeting cadence with each recruited health center, with most meetings taking place virtually. The data from site visits and meeting logs were utilized to devise community health center-specific project plans, which are not reported here to preserve the community health centers’ privacy.

### Data analysis

We computed descriptive statistics such as patient counts by demographic characteristics and diagnoses. The existence of hypertension policies was dichotomously captured as yes/no. In this pragmatic, service-delivery oriented project, data collectors coded their notes and qualitative data in real time during interviews labeling factors discussed as either facilitators or barriers to SMBP program implementation. Throughout the project, the team met weekly and engaged in iterative discussions to synthesize the rapidly coded data with data from other sources (e.g., site visits, meeting logs). The team integrated all data into a table detailing the stated and observed barriers and facilitators to SMBP implementation by health center.

## Results

All clinics (*N* = 6) reported serving more female patients than male patients (range: 55.0 to 71.6%). The proportions of Hispanic patients served by health centers ranged from 6.5 to 71.1%, and proportions of uninsured or self-pay patients ranged from 18.5 to 72.3% (Table 1).

**Table 1:** Health center and patient population characteristics.

Characteristics	Health center 1	Health center 2	Health center 3	Health center 4	Health center 5	Health center 6
Health center						
Health center type	FQHC	FQHC	FQHC	FQHC	FQHC	FQHC
Number of clinics	5	6	3	5	1	2
Patients						
≥18 years of age^1^	15,277	9,881	12,435	12,094	3,270	2,878
Gender						
Male	39.7%	41.1%	45.0%	23.2%	36.2%	38.7%
Female	59.7%	57.6%	55.0%	71.6%	63.8%	58.9%
Other^2^	0.1%	1.3%	0%	5.2%	0%	1.1%
Missing	0.5%	0%	0%	0%	0%	1.3%
Ethnicity						
Hispanic or Latino	49.2%	44.2%	17.6%	71.1%	N/A^3^	6.5%
Not Hispanic or Latino	36.4%	54.1%	81.3%	26.4%	N/A	92.4%
Missing	14.4%	1.8%	1.1%	2.5%		1.1%
Race						
White	10.4%	55.5%	90.8%	53.3%	16.7%	62.7%
Asian	6.5%	2.4%	0.7%	2.7%	1.8%	1.2%
African American/Black	18.0%	36.5%	6.6%	15.3%	7.8%	32.1%
Other^3^	2.8%	2.4%	1.9%	7.4%	2.6%	2.9%
Missing	62.4%	3.2%	0	21.2%	71.0%	1.1%
Insurance types						
Private insurance	24.8%	16.8%	53.3%	26.1%	N/A	21.5%
Medicare	3.2%	2.4%	12.6%	3.0%	N/A	12.8%
Medicaid	6.5%	8.1%	14.2%	20.8%	N/A	7.4%
Uninsured/self-pay	64.3%	72.3%	18.5%	50.1%	N/A	56.5%%
Other^4^	1.2%	0.4%	1.3%	0%	N/A	0.5%
Missing	0%	0%	0%	0%		1.1%
Comorbidities (*n*)						
Pre-diabetes diagnoses	3,242	796	N/A	328	N/A	546
Hypertension diagnoses	3,983	1,354	2,731	2,377	N/A	1,210

^1^Includes patients with ≥1 visit in 2019; ^2^Other includes transgender man, transgender woman, non-binary, genderqueer, and other; ^3^Indicates center was unable to provide values; ^4^Other includes American Indian, Alaska Native, Native Hawaiian or other Pacific Islander, and Other.

### Adoption and implementation barriers

Barriers to SMBP program adoption and implementation included: (1) limited funding and staffing; (2) limited tools to identify, treat, and manage patients; and (3) perceived limited patient engagement. Multiple leaders described funding limitations noting they could not procure reasonably priced blood pressure devices for at-home use which impacted their decisions to adopt an SMBP program. SMBP devices cost $37–$100 (14). Participants also described staffing shortages which would limit implementation. While all health centers reported existing processes to screen for hypertension, only two reported using clinical decision support tools to identify, treat, and manage hypertensive patients and to refer them to lifestyle change programs (Table 2). Finally, participants perceived challenges reaching patients and maintaining engagement in an SMBP program if implemented. They specifically described difficulties treating transient patient populations with whom they often lost contact making hypertension management difficult over time.

**Table 2:** Results from needs assessment survey (*N* = 6).

Factors	Clinic 1	Clinic 2	Clinic 3	Clinic 4	Clinic 5	Clinic 6
Hypertension identification and management						
Hypertension-related clinical decision support (CDS) tools						
We use CDS tools to identify patients with hypertension (HTN)	✓					✓
We use CDS tools to treat and manage patients with HTN	✓				✓	✓
We use CDS tools to help refer patients with HTN to community lifestyle change programs (LCP)	✓					✓
Screening						
Yes, we have a process for screening all adult patients for HTN	✓		✓	✓	✓	✓
Yes, we have a process for screening adult patients with risk factors for HTN (i.e., overweight, obese, family history etc.)		✓				✓
No, we do not have a process for screening adult patients for HTN						
Education						
Print materials are given to the patient	✓	✓	✓	✓		✓
The physician delivers verbal education	✓	✓	✓	✓	✓	✓
Non-physician team members (NPTM) provide verbal education	✓	✓	✓	✓	✓	✓
We do not provide education for hypertension						
Referrals to LCPs for hypertension						
Yes						✓
No	✓		✓	✓		
Not Sure		✓			✓	
Referrals to LCPs for Pre-diabetes/Diabetes Type 2 (DM2)						
Yes						
No	✓	✓	✓	✓		
Not Sure					✓	✓
Existing SMBP policies and practices						
SMBP Policy						
Yes	✓		✓			
No				✓	✓	✓
Not Sure		✓				
Recommendations to SMBP						
Never						
Rarely						
Some of the time				✓		✓
Most of the time	✓	✓	✓		✓	
Always						
Not Sure						
Provider reviews SMBP monitoring						
Always						✓
Most of the time	✓	✓	✓	✓	✓	
Some of the time						
Rarely						
Never						
Not Sure						
NPTMs are available to participate in hypertension management						
Yes	✓	✓	✓	✓	✓	✓
No						
Not Sure						
Patient portal capability						
Yes	✓	✓	✓	✓		✓
No						
Not sure					✓	
Percentage of people using the patient portal	2%	2%	n/a	70%	10%	54.30%
Use of telehealth						
Use of telehealth for chronic health management	DM2, HTN, CHOL, Mental Health, Nutrition	HTN, CHOL	DM2, HTN, CHOL	DM2, HTN, CHOL, all chronic diagnoses	Counseling	DM2, HTN, CHOL

DM2, Type 2 Diabetes Mellitus; HTN, hypertension; CHOL, cholesterol.

### Adoption and implementation facilitators

All community health centers reported conducting hypertension screenings and providing hypertension education to patients; although, hypertension policies and guidelines were not standardized (Table 2). Surveyed staff at recruited health centers were also familiar with SMBP programs. Two health centers had existing policies in place for providers to encourage patients to practice SMBP monitoring, although they did not provide blood pressure devices. Despite not having formal policies, the remaining health centers reported that providers recommended SMBP to patients at least “some of the time.” At all health centers, providers reviewed readings when patients chose to engage in SMBP and used the data to diagnose or manage patients with hypertension. Finally, all health centers reported having non-physician team members (e.g., nurse practitioners, medical assistants, patient care technicians) available to integrate into hypertension management practices, including implementing an SMBP program. For example, non-physician team members were responsible for rooming and recording a patient’s blood pressure during an appointment providing a potential opportunity for intervention.

### Adopters and implementers

Adopters (i.e., those responsible for adopting an SMBP program in the health center) included leadership and administrators, such as chief operating officers, nursing directors, care managers, and information technology operations analysts. These individuals had decision-making capacity within each health center. Implementers (i.e., those tasked with integrating an SMBP program into clinical protocols) included healthcare providers and non-physician team members.

## Discussion

SMBP is an evidence-based intervention that can help to accurately monitor a patient’s blood pressure in real time to successfully improve blood pressure control (7). In this study, we assessed baseline factors potentially influencing SMBP adoption and implementation in community health centers and identified key individuals in the health centers to support adoption and implementation. Importantly, the findings inform our understanding of how to best allocate resources to incorporate SMBP programs into clinical workflows, and they informed subsequent Implementation Mapping steps in developing and adapting implementation strategies to enhance SMBP adoption and implementation in health centers.

Many adopters recognized the importance of SMBP programs for patients, and providers often recommended it to their patients on a case-by-case basis. However, health centers did not have systematic approaches to implementing SMBP programs due to staffing constraints and lack of SMBP-specific policies to follow, factors that align with previous studies assessing barriers to SMBP implementation (5, 15, 16). This is consistent with literature suggesting priorities are often situational and context dependent in primary care settings (17). In these community health centers, home-based blood pressure monitoring may have been a lower priority compared to clinic-based monitoring for hypertensive patients given the resources needed to implement an SMBP program and ensure success (15). These resources include ensuring adequate staffing and funding, designing workflows to include SMBP, optimizing the EHR to incorporate SMBP recordings in patients’ charts, and utilizing quality improvement measures to ensure program integrity (5, 15, 16). Careful coordination and prioritization of resources are critical to ensuring program success (18).

This study has some limitations. Health centers self-selected into participating which potentially creates selection bias, and results may not be generalizable to all FQHC. In addition, recruitment happened in a large, urban metropolitan city in Texas limiting generalizability outside of the state and in more rural areas. Finally, we did not record or transcribe interviews with participants. This limited our ability to conduct thematic analysis to identify nuanced barriers and facilitators to SMBP adoption and implementation. Despite these limitations, findings informed the subsequent development of implementation strategies to increase SMBP program adoption and implementation in the participating health centers.

## Data Availability

The raw data supporting the conclusions of this article will be made available by the authors, without undue reservation.
